# Enhancement of Probe Signal for Screening of HIV-1 Protease Inhibitors in Living Cells

**DOI:** 10.3390/s121216759

**Published:** 2012-12-06

**Authors:** Huantong Yao, Sha Jin

**Affiliations:** Department of Biomedical Engineering, College of Engineering, 4188 Bell Engineering, University of Arkansas, Fayetteville, AR 72701, USA; E-Mail: hxy009@uark.edu

**Keywords:** molecular probe, FRET, HIV-1 protease inhibition, AcGFP1, mCherry, FLIM, high-content screening

## Abstract

The global human immunodeficiency virus infection/acquired immuno-deficiency syndrome (HIV/AIDS) epidemic is one of the biggest threats to human life. Mutation of the virus and toxicity of the existing drugs necessitate the development of new drugs for effective AIDS treatment. Previously, we developed a molecular probe that utilizes the Förster resonance energy transfer (FRET) principle to visualize HIV-1 protease inhibition within living cells for drug screening. We explored using AcGFP1 (a fluorescent mutant of the wild-type green fluorescent protein) as a donor and mCherry (a mutant of red fluorescent protein) as an acceptor for FRET microscopy imaging measurement of HIV-1 protease activity within living cells and demonstrated that the molecular probe is suitable for the High-Content Screening (HCS) of anti-HIV drugs through an automated FRET microscopy imaging measurement. In this study, we genetically engineered a probe with a tandem acceptor protein structure to enhance the probe’s signal. Both *in vitro* and *in vivo* studies revealed that the novel structure of the molecular probe exhibits a significant enhancement of FRET signals, reaching a probe FRET efficiency of 34%, as measured by fluorescence lifetime imaging microscopy (FLIM) measurement. The probe developed herein would enable high-content screening of new anti-HIV agents.

## 1. Introduction

Human immunodeficiency virus (HIV) was discovered in the early 1980s and has been responsible for an estimated 25 million deaths. Today, approximately 33 million people worldwide live with HIV, including one million people in the United States. If untreated, HIV causes acquired immunodeficiency syndrome, or AIDS. Currently, none of the anti-HIV drugs, even when combined, represent the ideal therapy due to their side effects, the high costs of treatment, and the emergence of drug resistant viruses [1,2]. Therefore, continuous effort is required to identify novel and effective drugs for HIV/AIDS therapy that exhibit improved safety and efficacy. HIV-1 protease is a small 99-amino acid aspartic protease that plays a crucial role in the viral life cycle and is essential to HIV replication [3]. This enzyme is required for the post-translational cleavage of HIV’s Gag, Gag-pol, and Nef precursor polyproteins and the conformational rearrangement of immature virions, which leads to the production of infectious virus particles [3,4]. Blocking protease activity will impair the maturation of HIV and inactivate its infectivity, even as it makes copies of itself. Thus, HIV protease can be a good target for therapeutic intervention.

Förster resonance energy transfer (FRET) has been extensively applied to not only protein-protein interactions but also the development of molecular probes that can track various molecular activities inside a living cell [5–8]. For instance, in our previous studies FRET has been utilized to generate a novel nanosensor to monitor glucose concentration real-time inside living cells [9–12]. However, the efficiency of a FRET probe for *in vivo* microscopy imaging measurement depends heavily on what is selected for the FRET pair as well as the structure of the fusion protein that constitutes the FRET probe. CFP (cyan fluorescent protein) and YFP (yellow fluorescent protein) have widely been used as a FRET pair for the microscopy imaging of molecular events within living cells. Due to the CFP/YFP pair’s crosstalk and photobleaching, which hinder the accuracy and reliability of CFP/YFP FRET probes in microscopy imaging measurement within living cells, an alternative FRET has recently been investigated [12–15]. It has been verified that a mutated EGFP-mCherry (a mutant of red fluorescent protein) shows ideal properties for FRET measurement and yields high accuracy both *in vitro* and *in vivo*[14]. On the other hand, the sandwich-like structure of FRET probe proteins, including the link in between the donor and acceptor proteins, play a critical role in the sensitivity of the FRET signal. The sensitivity of FRET-based nanosensors can be greatly improved by combining linker sequence optimization with fluorophore-insertion to decrease the degrees of freedom for fluorophore positioning [16]. Previously, to address these issues, we explored a new FRET pair in which AcGFP1 (a fluorescent mutant of the wild-type green fluorescent protein) served as a donor and mCherry acted as an acceptor for FRET microscopy imaging measurement of HIV-1 protease activity inhibitors within living cells [17]. We demonstrated that AcGFP1 is more tolerant of photobleaching, which makes quantitative analysis using AcGFP1 more reliable, and the molecular probe designated as GcC is suitable for high-content screening (HCS) of anti-HIV drugs through an automated FRET microscopy imaging measurement [17].

The efficiency of the FRET between a donor and an acceptor depends heavily upon the distance between them, and the characteristic Förster radius (R_0_) relies upon the alignment of both the donor and the acceptor’s fluorescence dipoles, the overlap of donor emission spectrum and acceptor excitation spectrum, the donor quantum yield, and the acceptor absorption coefficient [18]. Thus, it is necessary to optimize the alignment of AcGFP1 and mCherry fluorescence dipoles so that an optimal FRET efficiency can be achieved. In this study, we developed a molecular probe with enhanced signal sensitivity that can be used to visualize or quantitatively analyze HIV-1 protease inhibitors by screening a combinatorial library through HCS. We genetically engineered a probe with a tandem acceptor protein structure and designated it as GcCC. Both *in vitro* and *in vivo* studies demonstrated that the novel structure of the molecular probe exhibits a significant enhancement in FRET signals. The FRET efficiency of the probe developed herein increased greatly, as measured by fluorescence lifetime imaging microscopy measurement (FLIM).

## 2. Experimental Section

### 2.1. Construction of the Probe

AcGFP1 is a mutant green fluorescent protein with an excitation wavelength of 475 nm and an emission wavelength of 505 nm. mCherry is a red fluorescent protein with an excitation wavelength of 587 nm and an emission wavelength of 610 nm. The mCherry sequence was fused to the C-terminus of the mCherry to form a tandem structure of the fusion protein based on our previous molecular clone [17]. Briefly, mCherry was polymerase chain reaction (PCR) amplified from the plasmid, pmCherry (Clontech Laboratories, Inc. Mountain View, CA, USA) using the following pair of PCR primers. Forward primer: 5′-GTCGACGGATCCGTGAGCAAGGGCGAGGAGGAT-3′ and reverse primer: 5′-GAGCTC GGTACCCTTGTACAGCTCGTCCATGCC-3′. The deoxyribonucleic acid (DNA) fragments after PCR were inserted into the pTA-GcC [17] using BamHI and Kpn I restriction enzyme sites to generate an AcGFP1-p2/p7-mCherry-mCherry fusion protein with a (his)_6_ tag fused at its C-terminus. The resultant DNA plasmid was referred to as pTA-GcCC and used for *in vitro* studies. In addition, forward primer 5′-GTCGACGGATCCGTGAGCAAGGGCGAGGAGGAT-3′ and reverse primer 5′-GTCGACGGATCCGCGGCCGCCTTGTACAGCTCGTCCAT-3′ were used to PCR amplify mCherry gene, and the PCR product was subcloned into pLVX-GcC to generate pLVX-GcCC to allow fusion protein expression in mammalian cells. All expression vectors were verified by DNA sequencing analyses.

### 2.2. Purification of the FRET Probe

The production of the probe protein was carried out as described in our previous work with modification [17]. In brief, recombinant *E. coli* (New England Biolabs, Ipswich, MA, USA) harboring the pTA-GcCC that encodes the probe was grown at 37 °C until they reached an OD_600_ of 0.4∼0.5. Isopropylthio-β-galactoside (IPTG, 1 mM) was then added to the culture medium to induce the probe’s expression, and the recombinant *E. coli* was cultured at 30 °C for 4 h. The bacteria were harvested by centrifugation at 3,000 rpm for 15 min and the pellets were stored at −20 °C until use. The pellets were lysed in a phosphate buffer formatted bacterial protein extraction reagent (Pierce, IL) containing 1 mM phenylmethanesulfonyl fluoride. The lysate was filtered through a 0.45 μm filter (Millipore, Billerica, MA, USA) before it was loaded onto a Ni-charged immobilized metal affinity chromatography (IMAC) column (BioRad, Hercules, CA, USA). The binding buffer contained 0.05 M KH_2_PO_4_, 0.3 M KCl, and 40 mM imidazole (pH of 7.4), and the probe protein was eluted from the IMAC column using a buffer containing 0.05 M KH_2_PO_4_, 0.3 M KCl, and 500 mM imidazole (pH of 7.4). The purified probe proteins were then dialyzed against the protease reaction buffer, which contained 0.8 M NaCl, 80 mM sodium acetate, 1 mM EDTA, and 1 mM DTT (pH 6.0). Sodium dodecyl sulfate polyacrylamide gel electrophoresis (SDS-PAGE assay) was used to determine the purity of the probe protein.

### 2.3. *In Vitro* Characterization of the Probe

The *in vitro* characterization of the FRET probe was conducted using a luminescence spectrophotometer (Perkin Elmer LS-55B, Perkin Elmer, Waltham, MA, USA). The fluorescent intensities of both AcGFP1 and mCherry were measured at 510 nm and 605 nm, respectively, by exciting the probe at 475 nm (the maximum excitation wavelength of AcGFP1). The slit width of both excitation and emission was set at 10 nm. The FRET signal was defined as a ratio of the fluorescent intensity of the acceptor mCherry over that of the donor AcGFP1 when excited at 475 nm and allowed for spectrum scanning from 490 nm to 650 nm.

To determine the probe function *in vitro*, HIV-1 protease (NIH AIDS Research & Reference Reagent Program) was added to a reaction buffer with a total volume of 60 μL at a pH of 6.0. The reaction buffer contained 0.8 M NaCl, 80 mM sodium acetate, 1 mM EDTA, 1 mM DTT, and 5 μg of the probe. HIV-1 protease’s digestion of the peptide’s cleavage site on the probe protein was performed at 37 °C for 1 h, after which the reaction was stopped by the addition of 40 μL of stop buffer (0.66 M potassium phosphate, pH of 8.0). To assess whether the probe can ascertain the inhibition of HIV-1 protease by an inhibitor, inhibitors such as ritonavir (NIH AIDS Research & Reference Reagent Program) were added to the aforementioned reaction along with the HIV-1 protease. The FRET signals generated from the probe in the presence and absence of inhibitors were measured and compared to determine whether or not the inhibitory effect on HIV-1 protease by inhibitors can be detected using the molecular probe. All of the measurements were carried out at room temperature in triplicate.

### 2.4. Transfection, FRET Analysis by Images Acquirement

Human embryonic kidney (HEK) 293T cells were cultured in Dulbecco’s modified Eagle’s medium (DMEM) with 10% heat-inactivated fetal bovine serum and penicillin-streptomycin. Cells were seeded in the wells of a six-well plate and cultured at 37 °C with 5% CO2 incubator overnight. The probe-encoding plasmid and HIV-1 protease expression plasmid were transiently transfected into the 293T cells using a transfection reagent from QIAGEN (Valenci, CA, USA). Expression of the probe protein was detected 50 h after transfection using inverted fluorescence microscope (IX71 Olympus, Tokyo, Japan) and Slidebook software as described elsewhere[12,17]. Eight regions were randomly chosen to calculate the average value of the FRET signal.

### 2.5. Western Blot

At 50 h post transfection, cells were harvested by centrifugation at a speed of 300 g for 5 min. The cells were then lysed with a lysis buffer containing 0.05 M Tris-HCl (pH 7.4), 1% Triton X-100, 0.1% SDS, 0.15 M NaCl, fresh made 1 mM DTT, and 1mM Phenylmethanesulfonyl fluoride. Western blot analysis was performed as described in our previous work [17,19]. Protein extracts were subjected to 12% acrylamide gels at 200 V for 35 min, and then transferred to polyvinylidene difluoride (PVDF) membrane. Mouse anti-mCherry was used as the primary antibody and the HRP-conjugate anti-mouse IgG was used as the secondary antibody.

### 2.6. Fluorescence Lifetime Imaging Microscopy Measurement (FLIM)

FLIM measurement was performed using a FLIM system which (LIFA, Lambert Instruments, Roden, The Netherlands) consists of a signal generator, a 3 W light emitting diode (LED) used as an excitation source, an intensified charge-coupled device (CCD) camera with a multi-channel plate (MCP) based image intensifier, and an inverted phase contrast fluorescence microscope (IX51, Olympus). The LED and the signal generator are all computer controlled through LI-FLIM 1.2.7 software (Lambert Instruments). An LED light source at 485 nm, a filter cube comprising of a 470/40 nm band-pass excitation filter, a 495LP dichroic mirror, and a 525/50 nm band-pass emission filter (Chroma Inc, Lititz, PA, USA) were set to detect the lifetime of AcGFP1. Before performing FLIM, the cell culture medium was replaced with a PBS buffer. The lifetime of the AcGFP1 expressing donor protein alone as well as that of the donor-acceptor protein probes was acquired. Ten regions were randomly chosen to calculate the average value of the lifetime of each sample.

### 2.7. FRET Efficiency

FRET efficiency (E) was calculated by the Equation (1):

(1) $$E=\left(1-\frac{\tau_{DA}}{\tau_{D}}\right)\cdot 100\%$$

where τ_D_ is the lifetime constant of the donor protein in the absence of the acceptor protein, and τ_DA_ is the lifetime constant of the donor protein in the presence of the acceptor protein [20].

## 3. Results and Discussion

**Figure 1.**
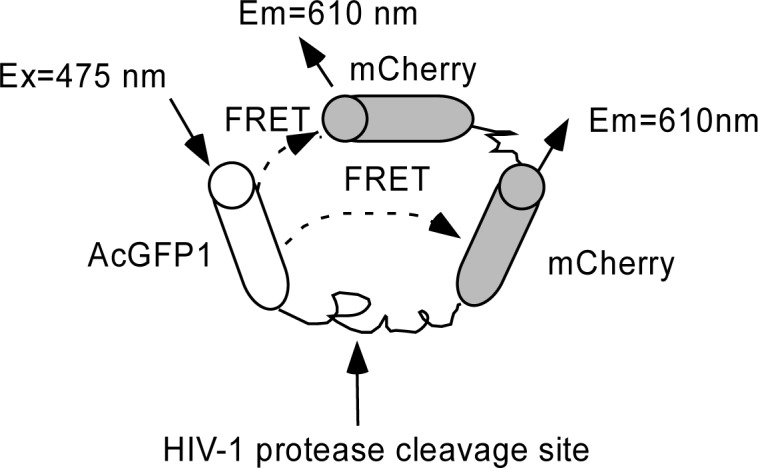
The schematic diagram of the FRET probe consists of tandem acceptors that function to enhance the absorption of energy resonance transferred from the donor. The donor AcGFP1 is linked with the acceptor mCherry through an HIV-1 protease cleavable peptide and the second acceptor is connected to the first acceptor.

We genetically engineered a FRET-based molecular probe with two acceptors in tandem, as shown in Figure 1, and the resultant probe was designated as GcCC.

We anticipated a shortened distance between the donor and acceptors of the FRET pair, and the two acceptors would lead to enhanced energy transfer efficiency, which will subsequently lead to an amplified FRET signal for screening anti-HIV protease agents. We determined whether or not the reporting capability of the probe can be improved by the tandem structure of the acceptor both *in vitro* and *in vivo*.

### 3.1. *In Vitro* Characterization of the Probe

**Figure 2.**
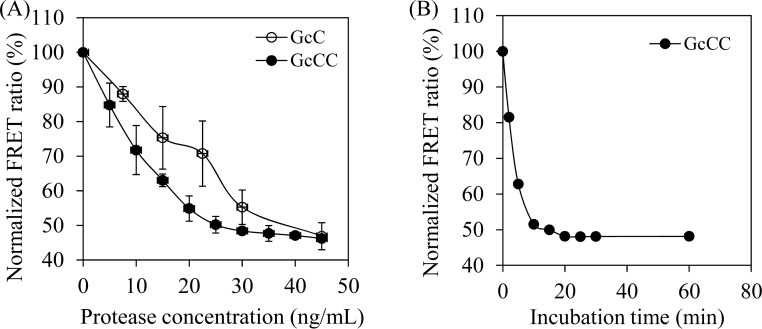
Characterization of the probes *in vitro*. (**A**) Effect of protease amount on the normalized FRET signal. 50 μg/mL probe protein were incubated with HIV-1 protease for 1 h. (**B**) Protease cleavage kinetics. 40 ng/mL of protease and 50 μg/mL of probe protein were used in the assays. Experiments were performed in triplicates. Symbols: GcC, a FRET probe with single acceptor protein; GcCC, a FRET probe with a tandem acceptor protein; Error bar: ±SD error.

The ability of the probe GcCC for reporting HIV-1 protease inhibition in live cells was characterized *in vitro*. The effect of HIV-1 protease concentration on normalized FRET signal was examined by adjusting protease concentrations up to 45 ng per mL in the reaction buffer, while the concentration of the probe protein GcCC was held constant at 50 μg per mL. The FRET signal value decreased in response to the increase of protease concentration in the range of 0 to 45 ng per mL, indicating the cleavage of the probe protein by HIV-1 protease (Figure 2(A)). The protease cleavage is concentration-dependent. Interestingly, compared to a probe harboring a single acceptor protein GcC, the probe harboring a tandem acceptor protein structure is more sensitive to a protease concentration range from 0∼35 ng/mL. A study on the effect of enzyme reaction time on FRET signal suggested that the protease cleavage reaction can be completed within 20 min (Figure 2(B)).

**Figure 3.**
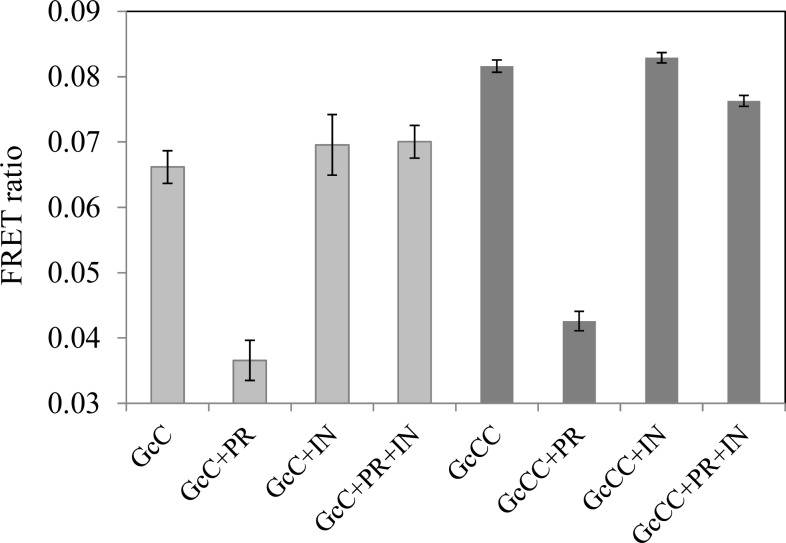
Detection of HIV-1 protease’s inhibition using the FRET probes. The reaction was conducted at 37 °C for 30 min and stopped by the addition of a stop buffer to the reaction buffer. All measurements were performed at room temperature. Symbols: GcC, a FRET probe with single acceptor protein; GcCC, a FRET probe with a tandem acceptor protein; PR, protease; IN: inhibitor. All experiments were performed in triplicate and the error bars stand for standard deviations.

To confirm the probe’s enzyme-specific decrease in FRET signal, the FRET signals were measured by adding the protease inhibitor ritonavir to the solution’s mixture. The absolute values of FRET signals obtained using GcCC and GcC are presented in Figure 3. We obtained higher absolute values of FRET signals using the tandem structured probe compared to using the probe GcC, which has a single acceptor protein (Figure 3: GcCC *vs*. GcC). The GcCC probe is not cleavable by the protease in the presence of ritonavir as FRET signal remains the same as that in GcCC alone (Figure 3: GcCC+PR+IN *vs*. GcCC). As a negative control, the probe signals did not change in the presence of the inhibitor without HIV protease (Figure 3: GcCC+IN *vs*. GcCC). Taken together, the experimental results indicated that the incubation of the probe with the HIV-1 protease will not quench the probe’s FRET when the protease activity is inhibited by its inhibitor. Thus, the decrease of the probe GcCC’s FRET signals in the presence of HIV-protease is enzyme-specific.

### 3.2. Enhanced Probe Signal for Detection of HIV-1 Protease Inhibition within Living Cells

**Figure 4.**
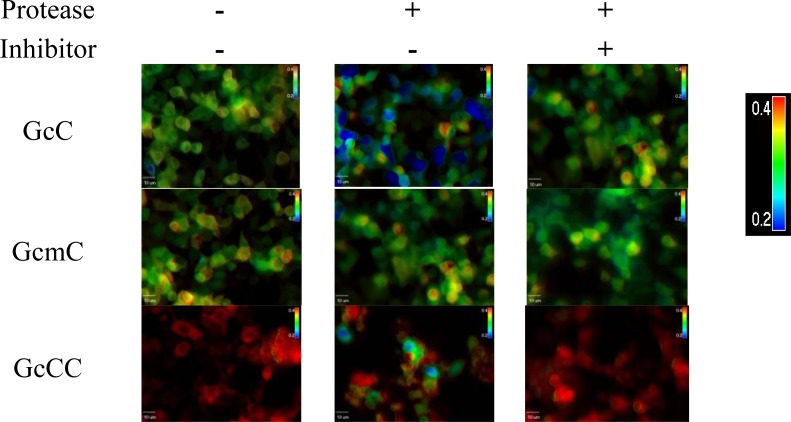
Visualization of HIV-1 protease activity and inhibition *in vivo.* 293T cells were either transfected with different structured probes alone or co-transfected with the probe and HIV-1 protease expression plasmids. FRET imaging microscopy measurement was performed at 50 h post transfection. Ritonavir, an HIV-1 protease inhibitor, was added to the cell culture medium to prevent the cleavage of the probe protein. The color bar indicates FRET ratio.

Having evaluated the detective ability of the probe GcCC to HIV-1 protease inhibition *in vitro*, we next examined whether the probe signal is indeed enhanced for visualizing HIV-1 protease inhibition within living cells. To this purpose, 293T cells were either transduced with the probe alone or co-transduced with both the probe and HIV-1 protease plasmids. FRET imaging microscopy measurement was performed to determine the probe’s FRET signals within living cells with our previous molecular probe GcC, which was constructed with one donor AcGFP1 to a single acceptor mCherry for comparison. Furthermore, to ensure the specificity of the enzyme reaction, a mutated probe, GcmC, in which a mutated cleavage site was constructed to make the probe non-cleavable by HIV-1 protease, was also employed for validation. As displayed in Figure 4, the probe GcCC and protease plasmid co-transfected cells in the absence of protease inhibitor displayed, reduced FRET signals at 50 h post transfection by showing mixture of blue, green, yellow, and red colored cells (Figure 4: GcCC with Protease+ and Inhibitor-) as compared to that signals produced from cells in the presence of protease inhibitor (Figure 4: GcCC with Protease+ and Inhibitor+). All of the cells displayed a red color if protease activity was inhibited by its inhibitor, indicating a FRET ratio of 0.4 (GcCC with Protease+ and Inhibitor+). The probe GcCC and protease co-transfected cells emitted sensitized red fluorescence when an inhibitor, ritonavir was added to the cell culture medium. The addition of the protease inhibitor to the cell culture medium prevented the cleavage of the peptide linker by the protease and thus retained the high FRET signal. As a control, the mutated probe has no response to either the presence or absence of the protease, and neither does it respond to the protease inhibitor, suggesting that the molecular probe designed in this study is specific to HIV-1 protease activity and inhibition.

**Figure 5.**
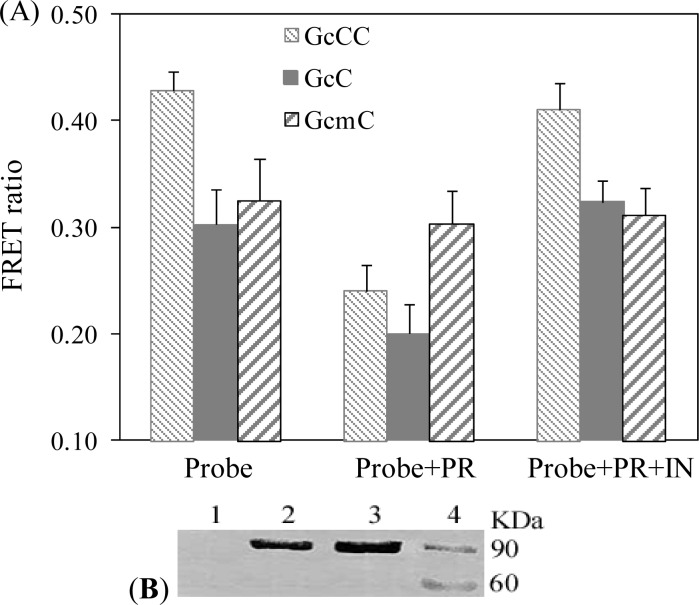
Enhanced signaling with tandem acceptors of the FRET probe *in vivo*. (**A**) Detection of the FRET signal in the presence or absence of protease inhibitor. Eight regions per well in a six-well plate were randomly selected for taking FRET signals. The average of the FRET ratios determined from these eight regions was used to represent the FRET signal of the probe in each well. Error bars show the standard deviation. (**B**) Confirmation of the cleavage of the probe protein by Western blot assay. Cells were harvested at 50 h post transfection and lysed for Western blot. Lane 1: 293T; lane 2: probe plasmid transfected cells; lane 3: probe and HIV-1 protease plasmid co-transfected cells in the presence of ritonavir; lane 4: probe and HIV-1 protease plasmid co-transfected cells in the absence of ritonavir. Experiments were performed in three independent experiments.

In the single donor-acceptor structure of the FRET probe GcC, FRET ratio changed from 0.20 to 0.32 [Figure 5(A)]. By contrast, FRET ratio changed from 0.24 to 0.42 in single donor-tandem acceptor structure of FRET probe GcCC. Thus, amplification of FRET signal can be obtained with the newly developed probe GcCC as compared to the previous FRET-based probe GcC. To confirm the protease’s cleavage of the probe, western blot assay was carried out to characterize the size of the probe protein in the presence or absence of protease activity. As shown in Lane 4 of Figure 5(B), two bands sized at 90 Kilodalton (kDa) and 60 kDa were observed in GcCC and protease cotransfected cells, whereas no 60 kDa band could be detected in mock transfected, GcCC alone transfected, and GcCC plus protease expressing vectors cotransfected cells in the presence of the inhibitor [Figure 5(B) Lanes 1–3]. Clearly, the presence of ritonavir in the cell culture medium prevented the cleavage of the probe (Figure 5(B), Lane 3), confirming the specific cleavage reaction for the probe detection system *in vivo*. It should be pointed out that with transient transfection as shown in Figures 4 and 5, it only took about 24 h of culture to detect the inhibitors’ inhibition of the HIV life cycle. We cultured cells for another 24 h to allow high protein expression levels of both probe protein and protease. At 2 days post transfection, significant protease inhibition can be visualized. As cells constitutively express probe protein and protease driven by a CMV (cytomegalovirus) promoter, this ascertains the stability of the two FRET-based probes for visualizing the inhibition of HIV-1 protease within living cells [17].

### 3.3. The Tandem Structure of the Probe Improves FRET Efficiency

**Figure 6.**
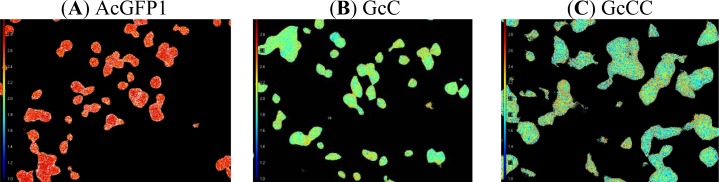
Fluorescence lifetime imaging of 293T cells expressing varied probe proteins. (**A**) AcGFP1 (donor alone), (**B**) AcGFP1-protease cleavage site-mCherry (GcC), and (**C**) AcGFP1-protease cleavage site-(mCherry)x2 (GcCC) lifetime image via FLIM. Lifetime lookup Table: 1.8–3.0 ns.

The lifetime of a fluorophore is solely dependent upon its local environment, such as a change in energy transfer from the donor to the acceptor [11]. Thus, changes in the lifetime of the donor lead to changes in the fluorescent probe’s FRET efficiency. To further evaluate the capacity of the developed probe, the lifetime of the donor fluorophore in different structured FRET-based probes was quantified using FLIM. We found that the lifetime of AcGFP1 detected from AcGFP1 alone expressing cells was 2.81 ns, whereas the lifetime of AcGFP1 measured in AcGFP1-cleavage-mCherry (GcC) probe expressing cells was 1.99 ns (Figure 6 and Table 1). The reduction of AcGFP1’s lifetime suggested the occurrence of FRET between the AcGFP1 and mCherry paired within living cells. With the tandem mCherry as acceptors in the FRET probe, the lifetime of AcGFP1 measured in AcGFP1-cleavage-mCherry-mCherry (GcCC) was 1.86 ns. The further reduction of the lifetime of AcGFP1 demonstrated clearly more fluorescence energy transfer from the donor to the acceptors, indicating enhanced FRET efficiency. Importantly, FLIM imaging revealed uniform FRET signal in the cells in all the cases tested (Figure 6), reflecting stability of the fluorophore AcGFP1. We calculated the FRET efficiency using Equation (1) with FLIM measurement since FLIM allows us to measure lifetime constants. As shown in Table 1, AcGFP1’s lifetime decreased significantly when the donor protein was fused with its acceptor protein, mCherry. AcGFP1’s lifetime was reduced by 29% with the presence of a single mCherry. Interestingly, with two acceptor mCherry proteins, the AcGFP1 lifetime was reduced by 34%, indicating that the fluorescence resonance energy transfer improved when using a tandem acceptor structure. The experimental result further confirmed that the new FRET probe designed herein possesses a high FRET efficiency.

Taken together, we focused on determining whether or not we could augment the probe signal to make the visualization of HIV-1 protease’s inhibition more sensitive by genetically remodeling a FRET-based fusion protein. We found a 4.7% increment in FRET efficacy with the GcCC probe, compared to an original value of 29.2% with the GcC probe, which is indeed a 16.1% improvement in FRET signaling. Further enhancement in this direction would be the adjustment of the linker sequences in between each protein. Indeed, fluorescent protein-based FRET efficiency highly depends upon the acceptor and donor pair as well as the linker sequences between the fluorophores, as reported by other groups [21–23]. We recently reported an AcGFP1-mCherry based glucose biosensor having a FRET efficiency of 22% for the real-time measurement of intracellular glucose concentration [11]. Its relatively low FRET efficiency is mainly due to the long sequences between donor and acceptor proteins and three dimensional structure of the fusion protein. In this study, we verified that, with the AcGFP1/mCherry pair, FRET efficacy can be improved by providing one more acceptor protein for further fluorescent energy transfer.

## 4. Conclusions

To enhance the sensitivity of the molecular probe for HCS of anti-HIV drug, a new probe has been developed by fusing AcGFP1 and a HIV-1 protease cleavable peptide and tandem structured mCherrys. Assessment of the characteristics of the probe developed in this study included FRET signal enhancement and FRET efficiency in the company of FRET and FLIM measurements. As expected, the reporting capability of the probe can be improved by optimizing its molecular structure. In both *in vitro* and *in vivo* studies, the new probe GcCC augments FRET signal, as compared to our previous probe, GcC. This indicates that the GcCC would be a better probe for drug discovery. Thus, the probe developed herein provides a useful and sensitive tool for visualizing protease inhibition within living cells and enables one to visualize HIV-1 targets within living cells for HCS of anti-HIV-1 protease inhibitors. Furthermore, we demonstrated that the GcC pair had a FRET efficiency of 29% and that a tandem structure of acceptor mCherry protein resulted in a FRET efficiency of 34%. The new FRET based probe structure developed here improved FRET efficiency. Taken together, our experimental results provide a strategy to enhance signal of a molecular probe that can be employed to a reliable and accurate high-content screening of anti-HIV-1 protease agents in living cells.

## Figures and Tables

**Table 1.** Average fluorescence lifetime of AcGFP1 from 293 T cells expressing AcGFP1, GcC, and GcCC.

	**AcGFP1**	**GcC**	**GcCC**
Fluorescence lifetime (ns)	2.81 ± 0.26	1.99 ± 0.21	1.86 ± 0.29
FRET efficiency (E)		29.2%	33.9%
